# When Helping Hurts: Children Think Groups That Receive Help Are Less Smart

**DOI:** 10.1111/cdev.13351

**Published:** 2020-01-03

**Authors:** Jellie Sierksma, Kristin Shutts

**Affiliations:** ^1^ VU Amsterdam; ^2^ University of Wisconsin–Madison

## Abstract

Helping has many positive consequences for both helpers and recipients. However, in the present research, we considered a possible downside to receiving help: that it signals a deficiency. We investigated whether young children make inferences about intelligence from observing some groups of people receive help and other groups not. In a novel group paradigm, we show that children (4–6 years) think groups that receive help are less smart (*n* = 44) but not less nice (*n* = 45). Children also generalized their inferences about relative intelligence to new group members (*n* = 55; forced‐choice‐method). These results have implications for understanding how children develop stereotypes about intelligence as well as for educational practices that group children according to their ability.

The exchange of help between social beings starts early in life (Warneken & Tomasello, 2006) and the positive consequences of helping have received tremendous attention in the social sciences. Receiving help can cause children and adults to succeed at their goals (Caprara, Barbaranelli, Pastorelli, Bandura, & Zimbardo, 2000; Newman, 2000; Zimmerman, 2002) and providing help makes people feel happy and valued (e.g., Aknin, Broesch, Hamlin, & Van de Vondervoort, 2015; Grant, & Gino, 2010). Furthermore, helping creates and maintains social bonds (e.g., Algoe, Haidt, & Gable, 2008), and is assumed to enhance the survival of groups (see De Dreu, Balliet, & Halevy, 2014; Warneken, 2018).

As some have noted, however, receiving help can also have negative consequences, especially because helping relations can be unequal: Recipients of help are often dependent on helpers who have more resources or control over a situation and therefore possess higher status. In such instances, a need or request for help could produce, perpetuate, or highlight inequality by making status differences salient (e.g., Glick & Fiske, 1997; Nadler, 2002). For example, receiving help might signal to bystanders that recipients of help are unable to accomplish a goal themselves.

Adults sometimes perceive those who receive help as less competent, especially when targets of helping belong to disadvantaged groups (Becker, Glick, Ilic, & Bohner, 2011; Nadler & Chernyak‐Hai, 2014). Whether receiving help signals to children that others are incompetent is not clear. Yet children, by virtue of their age, receive a great deal of help from others. Similarly, children often see other people receive help—for example, at school where competent individuals (i.e., teachers) regularly help those who are less competent (i.e., students). Understanding what helping signals to young children is important because their interpretation of helping could have consequences for academic and social development. For example, how children perceive and understand help might influence their own inclination to ask for help as well as how they perceive their peers.

Young children readily make inferences about others’ traits and abilities from observing how adults behave toward one another and toward children (e.g., Brey & Shutts, 2018; Castelli, De Dea, & Nesdale, 2008; Skinner, Meltzoff, & Olson, 2017). However, little research has investigated the inferences children make upon seeing adults provide help to others. As one exception, in research by Graham and Barker (1990), children observed a teacher who provided a hint to one boy on a math problem but did nothing for another boy. Six‐year‐old children (but not those who were younger) thought that the boy who received help was less smart than the boy who did not. Graham and Barker’s (1990) research provides initial evidence that children make inferences about competence—in this case, intelligence—based on the provision of help. However, the study was limited in scope (i.e., it focused solely on boys and math problems) and the design was not well balanced (i.e., a teacher helping was compared with a teacher walking by). It is therefore unclear whether the results were specific to receiving help or, for example, related to a teacher talking to and approaching one student but not the other.

Previous research has also failed to consider children’s inferences about receiving help in an intergroup context. Yet, just as some individuals receive more help than others, so, too, do some groups. Both adults (Cuddy, Fiske, & Glick, 2007) and older children (Sierksma, Lansu, Karremans, & Bijlstra, 2018) provide more help to people who belong to groups they think are incompetent. Moreover, schools across the world group children according to their competence (see OECD, 2013) and one consequence of being put in a lower competence group is that one likely receives, or is assumed to need, more help than a child in a higher competence group. Importantly, school groupings often align with students’ socioeconomic status (Batruch, Autin, Bataillard, & Butera, 2019) or racial background (Glock, Krolak‐Schwerdt, Klapproth, & Böhmer, 2013), and children therefore have opportunities to see that children from certain social backgrounds receive more help than others. As such, in addition to other well‐documented negative consequences of grouping children according to perceived or known ability (Van de Werfhorst & Mijs, 2010), tracking could also create a context that allows children to learn stereotypes about the competence of different groups.

Across three experiments we tested the hypothesis that children (4–6 years) perceive groups and group members who receive help as less competent than groups and group members who do not receive help. Perceived competence was assessed by asking children how intelligent they thought the groups and children were. Moreover, we focused on children ages 4–6 years, an age when children evidence robust and clear biases based on group membership (Dunham, Baron, & Carey, 2011; Raabe & Beelmann, 2011) and also have a rich understanding of competence and associated traits such as intelligence (e.g., Heyman, Gee, & Giles, 2003; Pasquini, Corriveau, Koenig, & Harris, 2007). We also presented novel groups (labeled and indicated by t‐shirt color) because children this age already have existing notions of how smart or nice, for example, particular ethnic or gender groups are (e.g., Bian, Leslie, & Cimpian, 2017; Roussos & Dunham, 2016).

## Experiment 1

Experiment 1 investigated the inferences that children make after observing some groups receive help and other groups receive no help. We predicted that children would evaluate the group that received help as less smart than the group that did not receive help. Given discussions about whether children’s evaluations of others are global or domain specific (i.e., halo effect; Koenig & Jaswal, 2011; Stipek & Daniels, 1990), we also sought to understand the specificity of the inferences that children make about those who receive help. In particular, we asked children how nice they thought the groups were.

All the experiments in this paper were approved by the ethical board of the university where the research was conducted and were preregistered at Open Science Framework (see https://osf.io/wams5 1, 2, 3). All data were collected in 2018 and are posted on OSF. All experiments were conducted with a different sample of children.

### Method

#### Participants

Our preregistered target sample size was based on Cohen’s (1992) recommendations, resulting in a minimum of 45 participants per cell for an 80% chance to detect a medium to large effect at an alpha level of .05 (expected effect size was based on piloting). As such, a total of 90 children were recruited. The final sample size was 89, however, because one child erroneously participated twice; data points for the second time were removed. Participants were between 56 and 81 months of age (*M* = 67.67, *SD* = 7.6; 45 boys, 44 girls). In each condition, children were the same age, *t*(87) = −0.59, *p* = .56. Testing took place at a preschool in a city in the Midwestern region of the United States and was performed by one of two trained research assistants who were unaware of the hypotheses. A total of 60 parents provided information on children’s racial background: most children were White (95%), and three children were multiracial.

#### Procedure

Children were tested in a room at their preschool where they wore headphones and were seated in front of a laptop. The experimenter told participants, “I want to show you some videos about groups of children. And you get to learn a little bit about these groups. And then afterward I have a question about it. OK?” Children watched three videos, answered one question about each video, and were then thanked and returned to their classroom.

#### Videos

Videos were constructed using the program Vyond (and can be seen at https://osf.io/wams5). Each video included animations of one of three activities where an adult expert provided help: solving a puzzle, doing a word game, or working on an art project. In each video, children saw two groups of children who were seated at a table. On top of this table were puzzle pieces, a computer, or art supplies (depending on the activity). The groups of children appeared to be talking and pointing at materials at the table; on each trial, both groups exhibited an equal amount of and similar movements. Each group consisted of four children from four racial groups and within each group children all wore clothes of the same color (i.e., green vs. red, blue vs. yellow, orange vs. purple). To maintain children’s interest and to distinguish the experts, we varied the apparent gender and race (i.e., White, Black or Asian) of experts across the videos.

Each video had the exact same structure and lasted 1 min and 16 s. For ease of exposition, we describe only the video about solving a puzzle here: Children saw two groups and the expert stood in the background equidistant between these groups. A female narrator introduced the groups, saying, “Here are two groups of children.” While the camera zoomed in on the group on the left, children heard, “Here are the *reds.* They all wear red. They are working on a really difficult puzzle.” Subsequently, the camera zoomed in on the group on the right and the same information was given with the exception that the color was different. Then, the expert was introduced: The narrator said, “And here is someone who knows a lot about puzzles.” After a short fade out, the expert then turned toward one group and looked at the group before saying, “I see you are doing a puzzle. Looks like you need help.” She then walked toward the group’s table while saying, “I’ll come help you” and stood next to the group’s table. After a short fade out, the children again saw the expert standing in the background between both tables. She then turned toward the other group and said, “I see you are doing a puzzle. Looks like you don’t need help.” She then walked toward the group’s table while saying, “I’ll come watch you,” and stood next to the group’s table. After a short fade out, participants saw a screen featuring just the two groups (no expert present).

We counterbalanced across trials and participants whether the expert first walked to the left or the right and whether she or he first helped or watched the group (i.e., sometimes the group on the left received help first and sometimes the group on the right side did; position of the color groups was kept consistent in the videos). In addition, the order of the activity presented in the videos (puzzle, word game, art project) was counterbalanced across participants. Condition (nice vs. smart) was randomized across children.

#### Perceived Intelligence or Niceness

For each video, children were asked how smart or nice they thought the groups were (between subjects). At the end of each video, with the groups of children still visible (but not the expert), the experimenter said, “So now I want to know how smart [nice] you think these groups of children are. So, do you think this group is smarter [nicer] [point left], this group is smarter [nicer] [point right] or are they the same?”. Children could provide their answer by pointing or telling the experimenter their answer verbally. To conduct *t*‐tests, children’s responses were coded as “−1” when they picked the group that received help as smarter, “0” when they said both were the same, and “1” when they said the group that did not receive help was smarter. Note that the preregistered analyses for age are reported in the Supporting Information.

### Results

**Figure 1:**
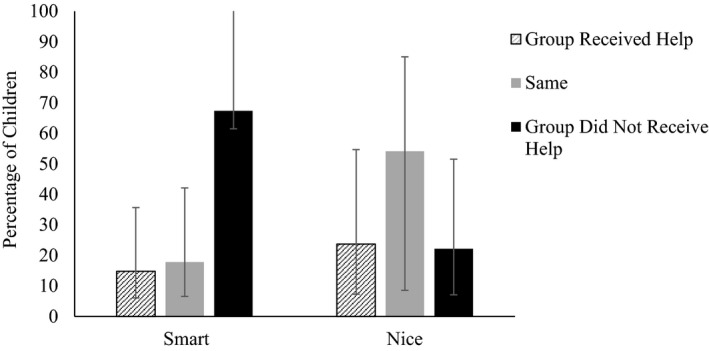
Children’s scores of how smart and nice the groups were according to whether they received help or not, Experiment 1, error bars represent confidence intervals (95%).

Figure 1 shows how often children picked each group or both groups in the smart and nice conditions. Chi square analyses showed that children’s responses differed from chance in the smart condition (χ^2^(2) = 70.71, *p* < .001) and in the nice condition (χ^2^(2) = 26.18, *p* < .001). *T*‐tests showed that the mean score across the three videos was above 0 in the smart condition (*M* = 0.52, *SD* = 0.61), *t*(43) = 5.58, *p* < .001, *d* = .84, but did not differ from 0 in the nice condition (*M* = −0.02, *SD* = 0.38), *t*(44) = −0.26, *p* = .80, *d* = −.03. Moreover, in the smart condition the mean score was significantly above the midpoint of the scale in all three domains of helping (i.e., puzzle, word‐game, art‐project, *t*‐tests, all *p*s < .001) and paired *t*‐test showed there were no differences between domains of helping (all *p*s > .38). Whereas the mean scores did not differ from the midpoint of the scale in the nice condition, suggesting a majority of the children rated the two groups as equally nice in all three domains of helping (*t*‐tests, all *p*s > .38) and there were no differences between domains of helping (all *p*s > .32). In addition, an independent samples *t*‐test showed that children’s scores in the smart condition were significantly higher than their scores in the nice condition, *t*(87) = 4.90, *p* < .001.

### Discussion

In line with our preregistered predictions, Experiment 1 provides evidence that a large majority of the 4‐ to 6‐year‐old children in our sample made inferences about a group’s intelligence from observing helping behavior. Specifically, a majority of the children inferred that the group that received help was less smart than the group that did not receive help. However, children did not perceive either group as nicer.

Given that endorsement of group stereotypes often leads to the application of stereotypes to individual members of the stereotyped group (Allport, 1954; Tajfel, 1981), Experiment 2 was designed to examine whether seeing groups receive help or not receive help would influence children’s evaluation of a new group member’s intelligence. Children often generalize their learning about novel groups to new members of those groups (e.g., Chalik & Rhodes, 2014; Horwitz, Shutts, & Olson, 2014). We therefore hypothesized that children would evaluate a new member of the group that received help as less smart than a new member of the group that did not receive help.

## Experiment 2

### Method

#### Participants

A preliminary version of Experiment 2 was conducted (*n* = 45) which revealed inconsistencies in how robustly children generalized their inferences about intelligence. However, some counterbalancing errors occurred in this preliminary study. We therefore decided to re‐run Experiment 2 with the errors corrected and with a larger sample size. Given the errors, we have chosen not to report the results here, but in the spirit of full transparency we have posted data for this study on OSF (see https://osf.io/wams5).

We tested 72 children between 48 and 83 months (*M* = 65.06 months, *SD* = 9.42; 31 girls, 41 boys). Parents provided information about children’s racial background; 63 children were White, four were Hispanic or Latino, one was Black, two were Asian, one was American Indian or Alaskan Native, and one did not report. Children were recruited from a child development laboratory’s database and testing took place at a university laboratory in the Midwestern region of the United States.

#### Procedure and Videos

**Figure 2:**
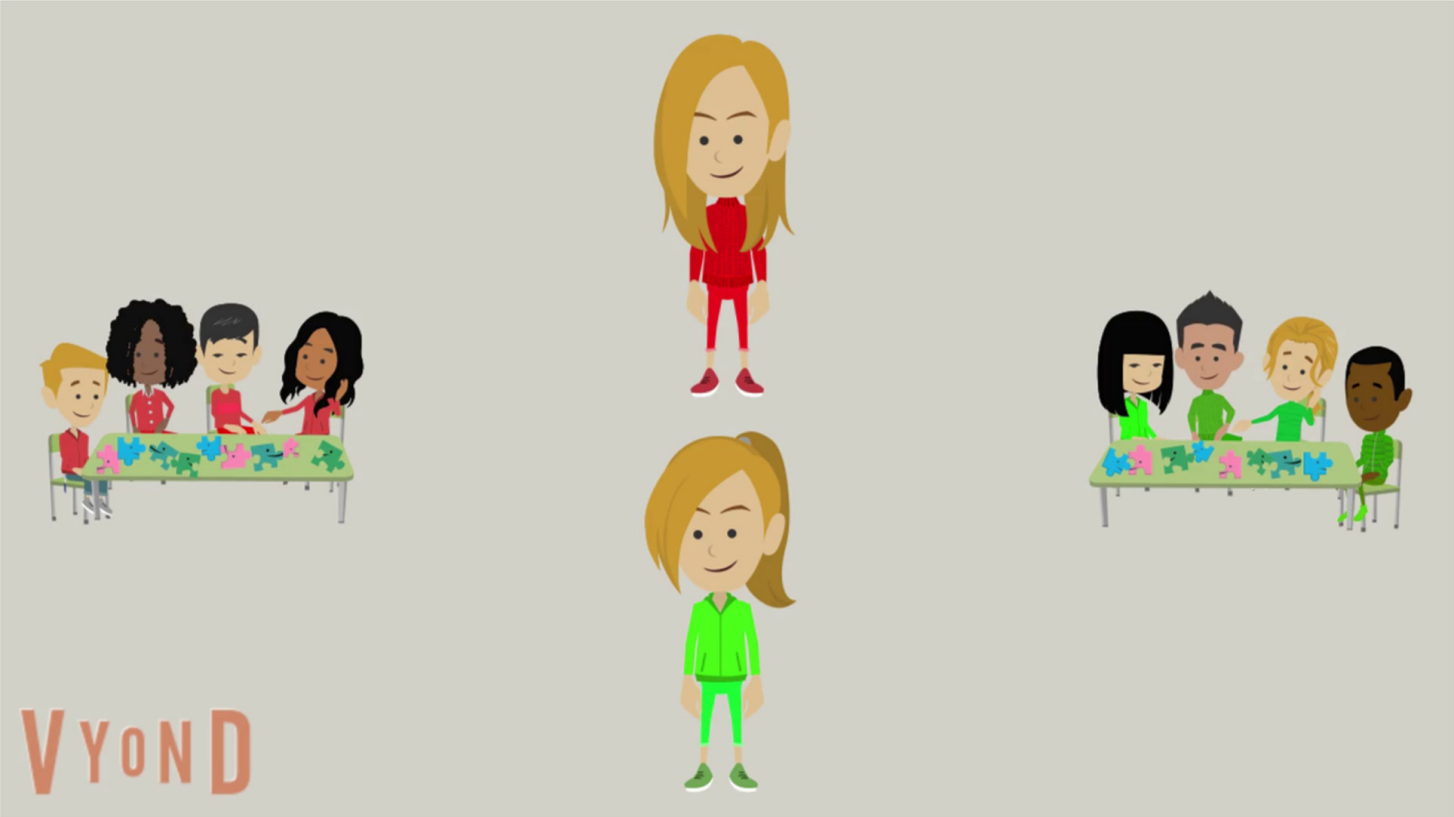
Last screen introducing new children, Experiments 2 and 3. [Color figure can be viewed at wileyonlinelibrary.com]

Testing was conducted by one of two trained research assistants who were unaware of which group received help and which group did not (children wore headphones so that experimenters could not hear what the narrator said). The procedure and counterbalancing were identical to Experiment 1, except that at the end of each video, participants were shown two new children (see Figure 2).

These new children were of the same gender and racial group as one another and solely differed based on t‐shirt color. These two children appeared in the middle of the screen, one above the other, at equal distance from the two groups of children. One of the new children wore the same t‐shirt color as the group on the left and one wore the same color as the group on the right (counterbalanced, as in Experiment 1). While these new children were shown, a female narrator said, “Here are two children you have not seen before.” For each video, children were then asked, “So now I want to know how smart you think these children are. So, do you think this child is smarter (point above), this child is smarter (point below) or are they the same?”. Pointing to a child that belonged to the group that received help was coded “−1,” pointing to both was coded “0” and pointing to the child who belonged to the group that did not get help was coded “1.”

Counterbalancing for where the expert walked first, whether the expert helped or watched first, and the order of the videos was the same as in Experiment 1. In addition, we varied across children the gender and apparent race of the expert and the new group members. Some children viewed only Asian experts and Asian group members for all trials; others viewed only White or only Black experts and group members.

### Results

Across all ratings the most frequent answer children gave was that both new children were equally smart (39.8% of responses), which was followed by saying the peer who belonged to the group receiving no help was smarter (34.7% of responses), and then that the peer who belonged to the group that received help was smarter (25.5% of responses). Although a chi‐square test suggested this distribution differed from chance (χ^2^(2) = 6.86, *p* = .03), a one sample *t*‐test showed that children’s ratings of how smart the new group members were did not differ from the midpoint of the scale (*M* = 0.09, *SD* = 0.55), *t*(71) = 1.42, *p* = .16, *d* = .16). Children’s scores also did not differ from the midpoint when we explored the results for each helping domain separately using *t*‐tests (i.e., puzzle, word‐game, art‐project, all *p*s > .14). In addition, paired *t*‐tests showed there were no differences between domains of helping (all *p*s > .43). Thus, most children did not indicate that new group members belonging to groups that had previously received help were less smart than new group members belonging to groups that had not previously received help.

### Discussion

Experiment 2 showed that a majority of the children did not think that new members of the group that had previously received help were less smart. Thus, most children did not generalize what they learned from observing one group receive help and another not receive help to new group members. On the one hand, this might mean that children truly inferred that both new group members were equally smart. On the other hand, however, these results might reflect that children were somewhat unsure about their inferences. After all, we only provided them with one instance in which groups received help or no help. To better differentiate between these two explanations, we conducted a third experiment in which we used a more sensitive test (i.e., forced choice, eliminating the “both” option). If children truly do not generalize what they learned about particular group members to new group members, results should replicate Experiment 2 (i.e., children should not indicate that new members of the group that had previously received help are less smart). However, if children were simply not that sure about whether to generalize their inferences, this more sensitive test should eliminate some of that uncertainty, perhaps resulting in generalization of their inferences to new group members.

## Experiment 3

### Method

#### Participants

Piloting suggested a larger effect size than Experiment 2 and we therefore recruited a slightly smaller sample size. A total of 57 children were tested. Two children were excluded (one child refused to pick either peer, one child’s parents interfered). Our final sample consisted of 55 children between 48 and 81 months (*M* = 63.93, *SD* = 8.80). There were 21 girls and 34 boys, and 45 children were White, seven were Hispanic or Latino, and three parents did not report racial background. Testing took place in a child development laboratory (*N* = 43) and preschool (*N *= 12) in the Midwestern region of the United States.

#### Procedure and Videos

We used the same videos and procedure as in Experiment 2.

After watching each video, the experimenter said, “So now I want to know how smart you think these children are. So, do you think this child is smarter (point above) or this child is smarter (point below)?”. Pointing to the new group member when he or she belonged to the group that received help was coded “−1” and pointing to new group member who belonged to the group that did not get help was coded “1.”

### Results

In 69.1% of the cases children said that a new group member belonging to a group that did not receive help was smarter, whereas in 30.9% of the cases did children say that the new group member belonging to the group that received help was smarter. A chi‐square test showed children’s responses differed from chance (χ^2^(1) = 24.06, *p* < .001). A one‐sample *t*‐test showed that the mean (*M* = 0.38, *SD* = 0.66) differed from the midpoint of the scale (i.e., 0), *t*(54) = 4.26, *p* < .001, *d* = .58. This pattern was evident across all three helping domains, albeit marginally for the art‐project video (binomial tests: puzzle, *p* = .001; word game, *p* = .003; art‐project, *p* = .058) and when we compared the domains of helping, no differences were found (paired *t*‐test, all *p*s > .22). Thus, when a forced‐choice format was used, children generalized what they learned from observing intergroup helping to new group members.

## General Discussion

Helping others has a wide range of positive consequences for both helpers and their recipients (e.g., Aknin et al., 2015; Algoe et al., 2008; Caprara et al., 2000; De Dreu et al., 2014; Grant, & Gino, 2010; Newman, 2000; Warneken, 2018; Zimmerman, 2002). However, receiving help can also have negative consequences: Here we show that a large majority of young children think that groups who receive help are less smart. The current research thus provides evidence for the idea that helping can serve as a social signal to children, supporting the formation of biased inferences about groups. That children as young as 4 years make these inferences underlines how powerful observing differential helping could be in guiding children’s view of social groups.

The inferences most children made from observing differential helping were specific to a group’s intelligence and not representative of a general inclination to view groups who receive help negatively; indeed, most children in Experiment 1 thought the groups were equally nice. Moreover, children’s inferences went beyond the specific group members involved in the receipt of help because children expected individuals who were members of a group that received help to be less smart than individuals belonging to a group that did not receive help (albeit only when we used a forced‐choice format; see below). In addition, we found evidence for the presence of these inferences across three helping scenarios (a puzzle, an art‐project and a word‐game).

We only found evidence for generalization when we used a forced choice format in Experiment 3. Why might this be? One possibility is that children were not that confident in their inferences (Experiment 2) and therefore a more sensitive measure was needed to capture children’s inferences (Experiment 3). After all, we provided children with a limited evidentiary base for their generalizations (i.e., only one instance where the group received help). Indeed, research on children’s personality inferences shows that young children sometimes do not make trait inferences based on one example of a target’s behavior (Boseovski & Lee, 2006). Importantly, this finding also suggests that observing one instance of differential intergroup helping does not automatically lead children to confidently conclude that a group receiving more help is less smart. Future research should seek to understand how and when observing intergroup helping renders stronger inferences, for example, by providing children with more instances of differential helping across groups.

It will also be important in future research to further understand the robustness and scope of the inferences children make about receiving help. For example, in real life, children’s experiences with observing others receive help might be “noisier,” such that they often see individual group members receive help or no help rather than groups as a whole. Future work should address if this variation affects children’s learning about a group’s intelligence and when and how this emerges in development. Related, going beyond novel color groups, future studies should test children’s inferences about existing groups in their communities. Additionally, in this study we chose to have experts deliver unsolicited help and it remains an open question if similar inferences would be made when help is *sought* by the group or its members. Another important avenue for future research is to address whether the provision of help could lead to the formation of other stereotypes. Although our findings indicated some specificity in children’s inference (i.e., intelligence, not kindness) future work should address if children make other kinds of trait inferences when in other domains such as athletic ability (i.e. receiving help or no help with sports) or wealth (i.e., providing resources to some but not others). Moreover, future research is needed to determine the conditions that might lead children to make positive inferences about receiving help. For example, it is possible that a recipient seeking or receiving help on a difficult task would signal competence rather than incompetence to children. Moreover, it is also possible that setting a clear norm that asking for help is beneficial or positive, would change children’s inferences about those who are the recipients of help.

Of course, adults are often interested in helping children and adults’ help improves children’s lives in various ways. Ability grouping, for example, is an educational practice that is implemented across the world (OECD, 2013) with as main aim to help children of all levels acquire academic success. However, by creating groups of children based on their competence, these practices also set the stage for group‐based helping. As such, tracking may ironically contribute to the perpetuation of inequality as children observe and make inferences about group members’ competence. Even in cases where children are not tracked by ability, adults’ differential helping can function as a social signal to children eager to learn about their social world. Given that adults’ own stereotypes about the intelligence of different groups (e.g., Fiske, Cuddy, Glick, & Xu, 2002; Zou & Cheryan, 2017) may lead them to help some groups more than others (Cuddy et al., 2007; Glick & Fiske, 1997; Sierksma et al., 2018), a self‐sustaining cycle might emerge in which adults help based on group stereotypes and subsequently children learn those stereotypes from observing them help.

## Supporting information


**Appendix S1.** Results for age.
