# Supplementary material for: When Helping Hurts: Children Think Groups That Receive Help Are Less Smart
Source: Child Dev. 2020 Jan 3;91(3):715–23. doi: 10.1111/cdev.13351 (PMC7244365; doi:10.1111/cdev.13351)
Supplement: Supplementary file 1 — Appendix S1. Results for age. [file CDEV-91-715-s001.docx]

**Supplemental materials**

For all studies we preregistered to explore how children’s age was related to their responses. Below we report the results.

**Study 1**

A regression analysis showed that children’s age (in months) was related to their responses about intelligence (*β* = .31, *p* = .04) but not their responses about niceness (*β* = .06, *p* = .69). We therefore conducted simple slope analyses and results showed that both older (1 *SD* above the mean) and younger (1 *SD* below the mean) perceived the group that did not receive help as smarter (younger: *b* = .33, *p* = .01*;* older: *b =* .73, *p* < .001).

**Study 2**

There was no significant correlation between age and children’s intelligence judgments (*r* = .04, *p* = .77).

**Study 3**

Mean rating did not correlate with age in months (*r* = .10, *p* = .48).
